# Explaining Alport syndrome—lessons from the adult nephrology clinic

**DOI:** 10.1007/s44162-024-00036-z

**Published:** 2024-05-13

**Authors:** Holly Mabillard, Rebecca Ryan, Nik Tzoumas, Susie Gear, John A. Sayer

**Affiliations:** 1 Renal Services, Newcastle Upon Tyne Hospitals NHS Foundation Trust, Newcastle Upon Tyne, UK; 2 Translational and Clinical Research Institute, Faculty of Medical Sciences, Newcastle University, Central Parkway, Newcastle Upon Tyne, UK; 3 Faculty of Medical Sciences, Biosciences Institute, Newcastle University, Central Parkway, Newcastle Upon Tyne, UK; 4 Sunderland Eye Infirmary, Sunderland, UK; 5 Alport UK, Cirencester, Gloucestershire, UK; 6 NIHR Newcastle Biomedical Research Centre, Newcastle Upon Tyne, UK

**Keywords:** Alport syndrome, X-linked, Kidney biopsy, Genetic testing, Collagen IV, Glomerular basement membranes

## Abstract

Alport syndrome is a genetic kidney disease that causes worsening of kidney function over time, often progressing to kidney failure. Some types of Alport syndrome cause other symptoms and signs, including hearing loss and eye abnormalities. Research now indicates that Alport syndrome (autosomal dominant inheritance) is the most common form. Alport syndrome can have X-linked or a rare form of autosomal recessive inheritance. Traditionally, a kidney biopsy was used to diagnose Alport syndrome, but genetic testing provides a more precise and less invasive means of diagnosis and reveals the underlying pattern of inheritance. At present, there are no specific curative treatments for Alport syndrome however there is a strong international effort in pursuit of future therapies. Currently, angiotensin-converting enzyme inhibitors (ACEi), or an angiotensin receptor blocker (ARB) if a patient cannot tolerate an ACEi, slow down the progression of kidney disease and can delay the onset of kidney failure by years. There are other potential treatments in research that potentially can help delay the onset of kidney issues. Early treatment of patients and identification of their at-risk relatives is a priority. People living with Alport syndrome and their doctors now benefit from an active international research community working on translating further treatments into clinical practice and providing up-to-date clinical guidelines.

## Introduction

This article is intended to educate kidney doctors and other doctors on Alport syndrome in adults, the holistic aspects of the disease, and the authors’ lessons learned from the adult kidney clinic. This article includes an Appendix ‘handout’ for patients and their families in addition to patient-centred diagrams, some less-technical explanations (labelled under ‘How it works’), and support group signposting intended for kidney doctors to use in the clinic setting to improve discussions with adults living with Alport syndrome. Appendix Table 8 includes a glossary of terms used throughout the text.

Alport syndrome is an inherited kidney disease that affects the glomeruli, is often progressive, and may be associated with sensorineural hearing loss and eye abnormalities. This article focusses on this collection of conditions called ’Alport syndrome’. However, there is a growing recognition that these are part of a ’spectrum' of conditions with a variable set of physical symptoms which include much milder conditions impacting the kidney compared with some of the more severe conditions that have symptoms that impact the kidney AND other parts of the body (Appendix Fig. 1).

Indeed, one of the major changes in the Alport syndrome community is the changing terminology of the range of diseases. From a genetic perspective, all these conditions are caused by variants in the collagen IV genes: *COL4A3*, *COL4A4*, and *COL4A5* [1, 2] collectively called the ‘Alport genes’. Conditions previously called ‘Thin Basement Membrane Nephropathy’ and ‘Benign Familial Haematuria’ are now termed by some experts in the field ‘Autosomal Dominant Alport syndrome’ even though their kidney disease is typically more mild and deafness is typically absent or occurs much later in life. Women and men with autosomal dominant Alport syndrome do not need to get unnecessarily worried by the term ‘syndrome’ as it is unlikely they will get the hearing or eye symptoms typically seen in the other genetic forms of Alport syndrome. The search continues for an appropriate term for a disease with large clinical variability.

Although classified as a ‘rare disease’, Alport syndrome is the most common genetic kidney disease in the UK and the second most common cause of kidney failure after autosomal dominant polycystic kidney disease [3, 4]. The population frequency is highest for conditions at the mild end of the spectrum, namely the autosomal dominant forms (at least 1 in 100). The population frequency is about 1 in 2000 for X-linked Alport syndrome and Autosomal Recessive Alport syndrome has an even lower population frequency [3, 4]. The core disease name ‘Alport’ comes from Dr Cecil Alport, a South African physician, who identified the inherited symptoms in a British family in 1927.

### Clinical course

The symptoms and signs associated with Alport syndrome and their severity in adults will depend on the type of causative variant (Appendix Table 1), its location along the collagen-IVa 3,4,5 trimer, the mode of inheritance (i.e. autosomal dominant, autosomal recessive, or X-linked) and potentially other factors, still to discover through research, such as whether ‘modifier genes’ impact severity creating different symptoms across members of a family who have the same genetic variant. Variants that have a substantial effect on the protein (e.g. truncations, frameshifts, large deletions/rearrangements) are generally associated with more severe disease, in comparison with milder variants (e.g. missense) [5, 6]. Men with X-linked Alport syndrome typically get more severe symptoms at a younger age, like that seen in men or women with autosomal recessive Alport syndrome; however, women with X-linked Alport syndrome can be equally severely affected so delays in diagnosis and treatment should not occur. The mean age at kidney failure for males with X-linked disease is 29 [1]. Men and women with a single autosomal variant and women with X-linked Alport syndrome show wider clinical variability and less predictable clinical outcomes (Appendix Fig. 1).

See HOW IT WORKS: Appendix Table 1. For a patient guide to the effects of genetic variants on protein production and likely clinical severity.

Men and many women with X-linked Alport syndrome in childhood or teenage years will typically have microscopic haematuria which often progresses as blood pressure increases. This weakens or damages the glomerular basement membrane and a decline in kidney function or, in some cases, kidney failure occurs. Most men with X-linked Alport syndrome, eventually require a kidney transplant or dialysis. The timing of when this happens is very variable. Sometimes, the uncertainty of ‘not knowing when kidneys will fail’ and having a rare condition that others do not understand is isolating and can cause mental health issues. It is important to direct people to mental health services and their national patient organisations so they get practical tips on how to cope and can connect with others in similar situations [7, 8].

Alongside chronic kidney disease (CKD), other features may occur (Appendix Fig. 2). Hypertension is very common and some people can get kidney cysts although these are typically asymptomatic. There is now a recognised link between *COL4A4* and *COL4A5* variants and kidney cysts [9]. Cysts can occur in any of the Alport syndrome subtypes but are much more likely to be found in those with proteinuria. Detected on ultrasound, cysts are typically few and small and are found in both the outer cortex and inner medulla of the kidney. They do not usually distort the kidney outline or increase kidney volume in contrast to autosomal dominant polycystic kidney disease (ADPKD) where cysts are often large enough to be felt externally and can cause pain [10, 11].

Like kidney cysts, the degree of proteinuria varies depending on the Alport syndrome subtype but this can vary greatly even within the same subtypes. Proteinuria can be present in all forms of Alport syndrome. In X-linked Alport syndrome, proteinuria usually develops in childhood (median age of 7 years for men and women). Proteinuria development is similar for both men and women with Autosomal Recessive Alport syndrome but typically occurs later in those with autosomal dominant Alport syndrome (median age 17 years) if at all [12]. Episodes of macroscopic haematuria can occur with intercurrent infections.

We must not ignore Leiomyomatosis which can occur although is extremely rare. This condition refers to smooth muscle tumours in various parts of the body and occurs particularly in the oesophagus, tracheobronchial tree, and female genital tract. This occurs in rare cases of X-linked Alport syndrome. The exact relationship between Alport syndrome and Leiomyomatosis is not fully understood but research is looking into this. Leiomyomas can be surgically removed if causing symptoms [13].

How Alport syndrome can affect the ears and eyes is detailed later.

See Appendix Fig. 2 (clinical features in Alport syndrome) and HOW IT WORKS: What do the kidneys do? For a patient guide to the function of the healthy kidney.

**Fig. 4:**
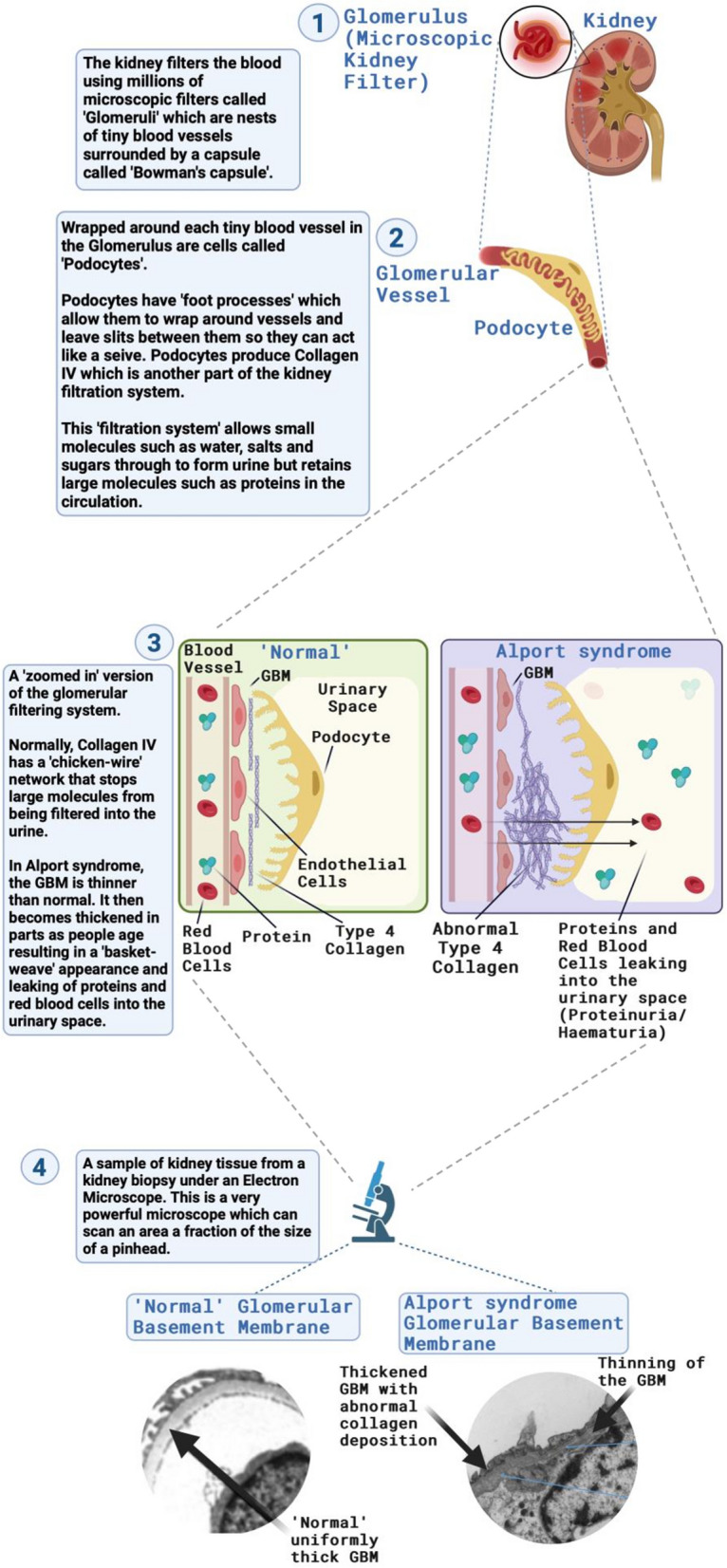
What happens in the kidney in Alport syndrome

See Appendix Fig. 3 (collagen genes to protein manufacturing) and Fig. 4 (what happens in the kidney in Alport syndrome) and HOW IT WORKS: The kidneys in Alport syndrome. For a patient guide to how the kidney does not function normally in Alport syndrome.

### How does Alport syndrome affect the ears?

Hearing loss is more frequent in people who have more severe Alport syndrome variants and hearing loss typically worsens with disease progression. People rarely go completely deaf and retain some hearing [14]. Ninety percent of men with X-linked Alport syndrome and 12% of women develop some degree of hearing loss by age 40; however, deafness is much less commonly reported in individuals with a single autosomal variant [15–17]. The collagen IV protein network plays an important role in the inner part of the ear (cochlea) and these proteins make up its basilar membrane, which allows the ear to detect high-frequency sounds. If there is even a small amount of hearing loss, hearing aids are essential and patients must be referred to audiology. Hearing loss typically affects a person’s ability to hear upper-register sounds such as children’s voices and background noise [18]. If hearing aids are not worn, patients permanently lose their neural pathways which reduces their ability to effectively use a hearing aid. Hearing loss also results in atrophy (shrinkage) within the brain’s higher auditory centres which complete development by age 16. This can result in lower-than-expected IQ development, poor academic performance, and negative effects on balance development, sports performance, speech development, speech discrimination in noise, attention, and social skills. Early intervention to protect hearing early in life is therefore critical.

See HOW IT WORKS: Appendix ‘How does Alport syndrome affect the ears?’ For a patient-centred explanation.

### How does Alport syndrome affect the eyes?

Since Alport syndrome is rare, and the eye changes do not always affect vision, it is difficult to know for certain how common the conditions listed in Appendix Table 3 are [19]. As the table illustrates most symptoms are treatable. Subtle changes, for example in the retina, may need specialist eye imaging to diagnose (Appendix Fig. 5). It is therefore important for people with Alport syndrome to have regular eye check-ups with an Ophthalmologist. This can ensure the maintenance of good vision. It is also important to treat other health problems such as diabetes and hypertension due to their effects on vision.

See Appendix Fig. 6 (basic anatomy of the normal eye and retinal changes in Alport syndrome).

One of the most common eye manifestations of Alport syndrome is the presence of yellowish flecks, known as’dot-and-fleck' retinopathy in the retina [19]. The retina is the light-sensitive tissue lining the back of the eye. It is not clear what causes these flecks, but they can extend to the blood vessels in the upper and lower parts of the retina and peripheral areas (Appendix Fig. 6). Although this retinopathy may become more noticeable with age, it does not affect vision or require treatment [19]. In some cases, these flecks can create a dull or less reflective area around the macula [20]. It is also very common to find that patients with Alport syndrome have a thinner area of the retina on the side of each eye closest to the ear compared with most people; however, this is not thought to cause any visual problems [21].

Macular holes, which cause blurred or distorted central vision are a rare complication of Alport syndrome, and if they occur, tend to be larger than those found in other conditions and may not respond well to surgical closure [19]. This is because people with Alport syndrome may have weaker neighbouring tissues, such as the internal limiting membrane, a transparent layer of tissue covering the surface of the retina [22].

Anterior lenticonus is a common feature of Alport syndrome that affects the lens of the eye. The lens is responsible for focusing light onto the retina [19]. This condition causes the front part of the lens to bulge outwards, giving it a cone-like shape. This can only be seen using a special instrument called a slit-lamp biomicroscope, where an’oil droplet' sign may also be seen (Appendix Fig. 7). As a result, people may experience visual problems such as blurred or distorted vision, and sensitivity to light. Anterior lenticonus is uniquely associated with Alport syndrome. Over time, the condition can worsen and affect vision, but lens replacement surgery can correct it.

See Appendix Fig. 7 (the ‘oil droplet’ sign and anterior lenticonus in Alport syndrome).

Alport syndrome can also affect the clear outer layer of the eye, called the cornea. Sometimes, people with Alport syndrome can get repeated scratches or damage to the top layer of the cornea, known as corneal erosions [19]. This can cause discomfort, pain, tearing, and sensitivity to light. While most cases of superficial corneal erosions heal on their own within a few days, more severe cases may require eye drops or ointments or specialist treatment.

Very rarely, a condition called posterior polymorphous corneal dystrophy (PPMD) can develop in the corneas of people with Alport syndrome, when they are children or teenagers. Sometimes, this can lead to cloudy or hazy vision and sensitivity to light [19]. However, most cases of PPMD are very subtle and do not need treatment [23], and only need to have the pressure in the eye monitored [24]. If people have corneal opacities in both eyes, corneal transplantation surgery may be an option, but only consider surgery if other treatments have not worked [24].

See Appendix Fig. 5 for real images of dot-and-fleck retinopathy and macular hole formation.

See HOW IT WORKS: Appendix ‘How does Alport syndrome affect the eyes?’ For a patient-centred explanation.

See Appendix Table 3 (checklist of eye signs to take to your Opthalmologist or Eye Surgeon).

### How is Alport syndrome inherited?

The three collagen IV genes involved in Alport syndrome are COL4A3, COL4A4, and COL4A5. COL4A5 is located on the X chromosome, and variants in COL4A5 cause X-linked Alport syndrome. COL4A3 and COL4A4 are both found on chromosome 2. Historically, the majority of cases of Alport syndrome diagnosed were predominantly X-linked inheritance with a few cases of Alport syndrome due to Autosomal inheritance. More recent studies show the proportion of Autosomal inheritance is much higher than originally thought [15, 17, 18, 25].

See Appendix Table 2 (Modes of Inheritance in Alport syndrome).

In X-linked Alport syndrome, an affected man typically inherits the condition from his mother who is ‘usually’ more mildly affected but, as previously mentioned, can be just as severely affected as a man (Appendix Fig. 8). In about 15% of men with X-linked Alport syndrome, the condition was not inherited from their mother but arises as the result of a new, or spontaneous (de novo) variant. Both men and women with X-linked Alport syndrome can pass the gene onto their children. A woman may pass the condition onto her sons or daughters and has a 50% chance of passing this onto their child regardless of sex. A man will only pass the condition to his daughters, never his sons, as he has to pass on his Y chromosome to have a son but all of his daughters will be affected. Boys inherit their X chromosome from their mother and it is important to offer genetic testing to the mother because of the 15% chance of their son’s variant being de novo.

For the other forms of Alport syndrome, where variants are found in the *COL4A3* or *COL4A4* genes, there are different patterns of inheritance:

- 1. In the autosomal recessive (AR) form of Alport syndrome, two faulty copies of the gene exist *‘in trans*’, one inherited from each parent, to develop Alport syndrome. AR Alport syndrome affects men and women equally and follows the same severe clinical pattern as seen in men with X-linked Alport syndrome (haematuria, proteinuria, kidney failure, retinopathy, and lenticonus). All the children of an individual with AR Alport syndrome will inherit one variant from their parent. Unless their other parent is also affected or has one faulty copy of the COL4 gene they should have a second normal copy of the gene. Autosomal recessive inheritance is less common in Alport syndrome, affecting about 1 in 40,000 individuals [26] and the family may be consanguineous. Often mistaken for X-linked disease, the impact on a family is very different as parents and offspring will not typically be affected and their siblings have a 1 in 4 chance of developing the disease.
- 2. People with one altered and one normal copy of an autosomal COL4A3 or COL4A4 gene have autosomal dominant* (AD) *Alport syndrome. This form of Alport syndrome was previously called ‘Benign Familial Haematuria’ or ‘Thin Basement Membrane Nephropathy’ and in some cases, it presents as ‘Focal Segmental Glomerulosclerosis’ (FSGS) where FSGS changes are apparent in kidney biopsy. People with autosomal dominant Alport syndrome have a greater risk than the general population of developing proteinuria and hypertension. A small number of these people with autosomal dominant Alport syndrome may see their kidney function decline. People with autosomal dominant Alport syndrome do not, however, usually develop any of the extra-renal features of Alport syndrome such as hearing loss or eye signs so some prefer to avoid the term ‘syndrome’ in this case. These people may, in later life, develop hearing loss for other, unrelated reasons. People who have a single autosomal variant have a 50% chance of passing this on each time they have a child, regardless of whether they are a man or a woman or whether they have a son or a daughter.
- 3. Rarely, Alport syndrome follows a compound heterozygous inheritance pattern otherwise termed ‘digenic’, where there are two disease-causing variants in different collagen IV genes, and this can result in a more severe form of the condition than if only one variant is present [27]. If these variants are on the same chromosome (‘in *cis’*) then this will be transmitted in an autosomal dominant pattern, and if they are on different chromosomes (‘in *trans*’) the inheritance pattern is autosomal recessive. Digenic Alport syndrome can also encapsulate those that have an autosomal (COL4A3 or COL4A4) variant AND an X-linked (COL4A5) variant.
- 4. De novo variants causing Alport syndrome mean that the variant has not been transmitted from the person’s parents and is ‘new’. The variant has occurred ‘spontaneously’ and has not been ‘inherited’ as DNA variants are always being caused by ‘chance’ when DNA is being replicated (usually in the sperm or egg or sometimes in the developing embryo). Although this would mean that the person with the de novo variant is the first person in their family line to have the variant, it can still be passed on to children and subsequent generations.

It should also be noted that modifier variants that affect other genes involved in podocyte and glomerular basement membrane function can worsen the clinical phenotype in Alport syndrome. In contrast, hypomorphic disease-causing variants (often missense variants or variants adjacent to the non-collagenous domains or interruptions of the collagen IV gene) can result in a milder phenotype further contributing to disease heterogeneity [28].

See Appendix Fig. 8 for a visual aid to understand X-linked inheritance in Alport syndrome with COL4A5 variants.

### Alport syndrome in women

Whilst X-linked Alport syndrome in women was previously thought to be a ‘benign’ form of the disease, research shows extensive variation within this group due to a random process of inactivation of one of the X chromosomes in each cell [17]. Every cell in a woman’s body has one X chromosome inherited from her mother and one from her father, but only one X chromosome is ‘activated’. This is a process that occurs randomly. Where the proportion of cells containing the ‘activated’ chromosome with the *COL4A5* gene variant is in abundance, the disease is more severe. This means a female with X-linked Alport syndrome can be affected with the full range of symptoms and signs and why the term ‘Alport syndrome carrier’ is problematic, dangerous, and no longer used. Women with X-linked Alport syndrome can have severe symptoms and should be monitored and treated in accordance with contemporary guidelines without delay.

Women carry a huge burden with this disease for a multitude of reasons including missed and delayed diagnoses due to being labelled as ‘carriers’. Twice as many women are affected by X-linked Alport syndrome as men due to the nature of X-linked inheritance. Whilst often having symptoms as severe as those seen in Autosomal Recessive Alport syndrome the number of affected family members is likely to be much higher in X-linked Alport syndrome due to the 50% chance of passing on the disease each time they have a child. X-linked Alport syndrome was previously described as ‘skipping generations’ but this is not correct and is simply a reflection of the undiagnosed women in the pedigree. It is important that X-linked disease is distinguished from autosomal recessive disease in women to dictate early initiation of treatment and aid screening of at-risk family members.

Fifteen to 30% of women with X-linked Alport syndrome develop kidney failure by age 60 [18, 25]. All women with X-linked Alport syndrome should be tested for hypertension and proteinuria annually at a minimum. Early genetic councelling must be offered in addition to clear information about their reproductive options and all women should have close monitoring throughout pregnancy, ideally under a medical obstetrics team.

All women should use contraception whilst taking an ACE inhibitor (or angiotensin receptor blocker (ARB)) due to the teratogenic risk in pregnancy of these medications. For women with any form of Alport syndrome, uncontrolled hypertension in pregnancy can exacerbate kidney impairment and proteinuria. Hypertension and kidney impairment both predispose a person to pre-eclampsia in pregnancy [29]. All women and men with any form of Alport syndrome who wish to have children should be informed that they can access prenatal diagnosis and preimplantation genetic diagnosis. This must be discussed early with all affected couples who would like to have children in the future so appropriate planning can be put in place.

### How is Alport syndrome diagnosed?

Getting a genetic diagnosis of Alport syndrome is very important, as this will enable treatment to be initiated earlier, which is associated with better outcomes. It also allows family members to be tested and treated sooner if needed. Enquiring about family history is both helpful in the diagnostic process (although not every person has a family history of kidney impairment or other extra-renal manifestations) and for determining the pattern of inheritance of Alport syndrome. It is also important to re-confirm if the clinical phenotype in a family segregates with the type of genetic variant found so that a 2nd variant is not missed, in the case of recessive or digenic disease, and appropriate family members are subsequently tested.

See Appendix Table 4 (When to consider a diagnosis of Alport syndrome).

In up to 30% of clinically suspected Alport syndrome, no pathogenic variant is found and this includes cases where there is a familial pattern of haematuria or thinning of the glomerular basement membrane on kidney biopsy [30, 31]. Sometimes a variant of unknown significance is found and some cases occur where the Alport syndrome is autosomal recessive or digenic but only one of the two variants is found. Table 1 (below) details how a genetic test report should be interpreted. These ‘missing’ variants occur because of incomplete coverage (especially of exons 5 and 6) of the studied region or difficulties in the detection of large gene deletions, Copy Number Variation, and non-canonical splice variants (variants that have a different genetic sequence to initiate splicing of introns than the common sequence) that affect gene splicing [32, 33]. Some of these missing variants are because the disease is a ‘phenocopy’ (a disease that clinically resembles Alport syndrome) [34]. Finally, synonymous variants (variants that do not change the amino acid sequence of the protein) or deep intronic variants can be missed and mosaicism can also occur (the cells within the same person can have different genetic makeup) [35, 36].

**Table 1:** Interpreting a genetic test report

It is not unusual for genetic testing to be negative. This does not mean that the individual affected does not have a genetic disease for the reasons described above
The reason for testing is specified such as ‘to investigate the cause of this patient’s renal phenotype’
There is usually a result summary where it will be made clear if the individual does or does not have a genetic diagnosis based on the type of test performed and the reason for testing. The gene change will be summarised here, whether the patient is homozygous or heterozygous and the disease associated with that genetic change if it is clear and known
The Online Mendelian Inheritance in Man (OMIM) code for the disease diagnosed is usually specified where this is available which provides further details of the clinical diagnosis
The implications of the result are specified such as the inherited risk to offspring and other relatives. A recommended action is also specified such as ‘testing of at-risk relatives after genetic councelling’
The gene name is in italics and the type of variant is described according to its genomic location, its location in the protein, and the type of variant it is, e.g. deletion, missense, and frameshift. The type of variant is important as this may influence access to participation in future clinical trials as well as a better understanding of their condition. All patients should therefore own a personal copy of their genetic test report
The variant will either be classified as pathogenic, likely pathogenic, a variant of uncertain significance (VUS), benign, or likely benign based on the ACMG scoring system [37]
For genetically undiagnosed individuals or those with a variant of uncertain significance, it is worth contacting the genetics laboratory (or in some cases repeating whole exome or genome sequencing) every 2–3 years as variants are being up or downgraded and new variants are being rediscovered all the time

In order to accurately diagnose these patients a multidisciplinary team is required including clinical geneticists, nephrologists, ophthalmologists, academics, and others. Extended genomic sequencing, RNA sequencing, bioinformatic tools, precise ocular phenotyping to look for Alport eye changes, and other techniques used to detect splice variants or possible mosaic diseases such as hair root or patient kidney cell analysis should be considered in genetically negative cases. Re-examining a kidney biopsy to look for lamellation in the patient or any affected family member may be helpful as well as genetic linkage studies if DNA is available from relatives with a known phenotype.

The importance of obtaining a genetic diagnosis goes beyond accurate labelling of the disease. It can indicate the mode of inheritance, allow for precision-based treatments as well as the avoidance of ineffective treatments, help identify at-risk family members (and potential suitable kidney donors), aid diagnosis of extra-renal involvement, aid family planning decisions and help determine the risks of rare but potential anti-glomerular basement membrane disease after kidney transplantation.

See HOW IT WORKS: Appendix ‘Getting a diagnosis of Alport syndrome’ for a patient-centred explanation.

Genetic testing is the preferred way to test for Alport syndrome. If a kidney biopsy is done, sometimes, staining for collagen IV is sometimes performed on the biopsy samples. Staining can, however, help with understanding the underlying genetics and outcomes of the condition. Those with severe Alport syndrome (for example, men with X-linked Alport syndrome and men or women with Autosomal Recessive Alport syndrome) typically do not have any alpha-3/4/5 collagen in the glomerular basement membrane compared with milder forms [5]. It is important to note that most laboratories only stain for alpha-5 collagen by immunofluorescence and may miss alpha-3 or alpha-4 chain deficiency. This must be checked by the requesting doctor so a diagnosis is not missed. A kidney biopsy may enable the exclusion of other glomerular and tubulointerstitial kidney diseases (e.g. interstitial nephritis, IgA nephropathy, or membranous nephropathy) when there is negative DNA analysis or an atypical clinical course. It should, however, be noted that the presence of a glomerular disease such as IgA nephropathy or primary membranoproliferative glomerulonephritis on biopsy does not exclude Alport syndrome as the abnormal glomerular basement membrane in Alport syndrome may predispose the glomeruli to immune-mediated injury [38, 39].

There is a small sub-group of people with Alport syndrome who present with a condition called Focal Segmental Glomerular Sclerosis (FSGS). They have heavy proteinuria and often oedema. Following a genetic test, the results may indicate a genetic variant in one of the Alport syndrome genes (*COL4A3*, *COL4A4,* or *COL4A5*) [40, 41]. This shows the importance of genetic tests to establish a precise diagnosis. The genetic tests also inform treatment decisions and can help to identify other family members at risk of Alport syndrome. In fact, COL4A variants in families with nephrotic-range proteinuria and variable degrees of haematuria often do not have all the classical features of Alport syndrome [42]. In one group, 2.5% of cases of steroid-resistant nephrotic syndrome had a COL4A variant [43]. Furthermore, COL4A variants were the most common significant genetic variant found in adults with FSGS (38% of familial FSGS and 3% of sporadic FSGS) [40]. A further study has shown that pathogenic FSGS gene variants were not commonly found on DNA analysis when Alport syndrome was clinically suspected [1].

It is important to explore and identify the underlying cause of people’s FSGS which could be Alport syndrome. A genetic test will ensure that people with FSGS get an accurate diagnosis and the appropriate genetic counselling. An accurate diagnosis using a kidney biopsy in this particular subgroup of people with FSGS emphasises the importance of performing electron microscopy when light microscopy of the kidney shows FSGS. This could reveal a thin glomerular basement membrane and prompt genetic testing of the person and their family for Alport syndrome. Indeed, high-dose corticosteroid treatment for a ‘presumed’ diagnosis of primary FSGS is not safe for an Alport syndrome patient, emphasising how genetic testing is really important to clarify appropriate treatment options.

### What treatment is available?

Currently, there are no curative treatments for Alport syndrome or any on-label Food and Drug Administration (FDA) and/or European Medicines Evaluation Agency (EMEA) approved therapies for Alport syndrome. However, the vibrant international research community is developing new treatments that will come into clinical practice. It is really important for families living with Alport syndrome and their nephrologists to review and understand the latest clinical guidelines, which are typically published after each of the international workshops on Alport syndrome [1]. The field is changing rapidly, so efforts to stay in touch with the latest guidance are imperative! Adults living with Alport syndrome should be under the care of a Nephrologist who will undertake a regular review of their kidney function and overall health.

Although not curative, inhibition of the renin–angiotensin–aldosterone system (RAASi) via ACE inhibitors (ACEi) or angiotensin receptor blockers (ARBs) can ameliorate the pace at which kidney function declines. It is suggested that a ‘the earlier the better’ approach to initiating ACE inhibition (or an angiotensin receptor blocker (ARB)) may be beneficial in Alport syndrome to maximise its benefits and extend the time kidneys function by more significant margins. In the event that a patient does not tolerate an ACE inhibitor due to a drug side effect, such as the development of a chronic cough, an angiotensin receptor blocker (ARB) should be used at the maximum tolerated dose. A multi-centre study in 2019 addressed this question, looking at the effect of this medication in children with Alport syndrome, hoping to commence treatment even before the process of fibrosis and scarring has set in. The results were encouraging, suggesting that early treatment with ACE inhibitors can slow the onset of kidney failure by many years [44]. It should be noted that renin–angiotensin–aldosterone system inhibitors are off-label treatments for Alport syndrome and thus not approved by the FDA or EMEA but are widely considered safe for Alport patients based on decades of real-world use.

Kidney function can decline more rapidly in certain situations so it may be necessary to start ACE inhibitors (or an angiotensin receptor blocker (ARB)) immediately at diagnosis, prior to the onset of protein in the urine (proteinuria) or obvious kidney function decline. These situations are summarised in Table 2 (below). For those who do not meet these criteria, this treatment should be initiated when there is persistent proteinuria [45].

**Table 2:** Genetic reasons to urgently initiate ACE inhibition (or ARB) prior to proteinuria onset

Situation	Meaning
All men with X-linked Alport syndrome	*Presence of a COL4A5 variant in a man*
All individuals with digenic Alport syndrome	*A variant in any 2 of the 3 COL4 genes*
All individuals with autosomal recessive Alport syndrome	*Two variants in the same COL4 gene (homozygous variants)*

Ultimately, some people reach kidney failure as young adults and need to understand their options for dialysis and/or a kidney transplant. As the majority of patients with Alport syndrome are otherwise fit and well, most are suitable for a kidney transplant. However, not all patients have suitable or readily available live donors and some may need to wait for a deceased donor kidney and require a period of dialysis. Alport patients have comparable dialysis outcomes to those with other non-Alport causes of kidney disease and both haemodialysis and peritoneal dialysis can be considered.

See Appendix Table 5 ‘Optimising your physical and mental health in Alport syndrome’.

### Kidney transplantation

Most people with Alport syndrome who receive a kidney transplant do very well. Twenty-year survival of kidneys after a transplant was significantly higher in Alport syndrome compared with those with other kidney diseases. The transplanted kidney survival was no different between those with Alport syndrome compared to other causes of kidney disease and was, in fact, better than age-matched controls without Alport syndrome in one study possibly due to the absence of other vital organ involvement in the disease [46]. A diagnosis of Alport syndrome does not need any adjustments to standard post-transplant immunosuppression regimens. Genetic testing of apparently healthy and unaffected family members who volunteer as potential live donors for a kidney transplant should begin as early as possible as they cannot donate a kidney if they have a COL3/4/5 variant. This is important as the benefits of a pre-emptive live donor kidney transplant mean that dialysis is avoided or minimised. It is possible to plan the surgery to reduce the risks involved, the new kidney usually works straight away, and the risk of complications from the new kidney is less as the the person who donates the kidney is likely to be healthier. A kidney from a living person (live donor kidney) usually lasts longer than a kidney donated from someone who died (deceased donor kidney) [47].

Since the advent of improved immunosuppression regimens in recent years, anti-glomerular basement membrane disease (anti-GBM disease) is now a very rare complication following kidney transplantation. Anti-GBM disease is traditionally thought to happen in people who have a whole gene deletion (usually in *COL4A5*), rather than a non-severe causative variant. The body’s immune system does not recognise the collagen chains present in the transplanted kidney because the gene was previously absent. As the collagen is unfamiliar to the body, an autoimmune attack of the donor kidney may occur. This process does not affect the lungs as seen in Goodpasture’s syndrome, a separate kidney disease involving antibodies to the glomerular basement membrane. This rare complication, if it develops, usually occurs within the first year after kidney transplantation, and the transplanted kidney loss develops within a few weeks to months in about 90% of these cases. However, in extremely rare cases, this complication can occur years after transplantation. Multiple treatment options are available to suppress the immune system, but it is a serious condition that can cause the transplant to fail [48].

An unrelated observation is the presence of isolated antibodies deposited along the glomerular basement membrane (linear IgG antibodies) on kidney transplant biopsies in patients with Alport syndrome. This is not to be confused with anti-glomerular basement membrane disease and it does not translate into poor transplant outcomes and can occur with any type of causative *COL4* variant [49].

### Potential future treatment options

There are ongoing trials looking into new treatment options aimed at slowing the disease course of Alport syndrome. A summary of potential therapeutics under investigation is listed in Table 3 (below) in addition to information on recently discontinued trials in Table 4 (below).

**Table 3:** Potential therapeutics under investigation for Alport syndrome

SGLT2 Inhibitors	40% reduction in urine protein creatinine ratio (uPCR) shown in a small and short prospective case series of 6 patients. Larger trials are needed but pre-emptive treatment already starting in clinical practice. (Clinical Trial Numbers NCT03036150, NCT03594110 and NCT01485978)
Finerenone	Finerenone is a more highly selective mineralocorticoid receptor inhibitor than others available such as Spironolactone. It reduces CKD progression and has cardiovascular benefits with fewer side effects than similar drugs. A mouse study has suggested that triple therapy with an ACE inhibitor, an SGLT2 inhibitor, and a mineralocorticoid receptor inhibitor such as Finerenone may substantially improve outcomes in Alport syndrome. At present, the FIONA trial is recruiting to investigate the effects of Finereone on the body when taken with an ACE inhibitor in children with CKD. (Clinical Trial Number NCT05196035)
Aminoglycoside analogues	A short and small safety-focused phase 1 trial using ELX-02 (an aminoglycoside analogue) was completed and a phase 2 trial is in progress for X-linked and autosomal recessive Alport syndrome with nonsense variants. The drug causes read-through of a stop codon to correct the nonsense variant and prevent premature termination of the peptide (Clinical Trial Number NCT05448755)
Endothelin type A antagonists	A Phase 2 Trial testing endothelin A receptor antagonists (endothelin A is upregulated in Alport syndrome) is in progress. Positive outcomes have already been found in mouse studies (reduced proteinuria, markers of kidney function, and improved kidney biopsy appearances). (Clinical Trial Number NCT04573920)
Hydroxychloroquine	Phase 2 trials are in progress in X-linked Alport syndrome testing this drug which is shown to suppress inflammation pathways. (Clinical Trial Number NCT04937907)
Lipid-modifying drugs	Lipid accumulation has been shown in Alport syndrome and drugs that bind cholesterol have shown promise in mouse studies. A phase 2 trial is currently testing a lipid-modifying drug. (Clinical Trial Number NCT05267262)
Gene replacement therapy	One study of mice in Japan showed promise for exon-skipping therapy in Alport syndrome as has another study testing a transgene in mice. CRISPR/Cas9 gene editing requires more development at present, as one report showed a few corrections to underlying gene variants as well as some undesired effects

**Table 4:** Recently discontinued trials in Alport syndrome

Anti-miR-21	The HERA Phase 2 trial tested inhibitors of micro-RNA-21 which have been shown to drive kidney fibrosis. Unfortunately, the trial was prematurely terminated due to no evidence of improvement in the rate of kidney function decline [50]. (Clinical Trial Number NCT02855268)
Bardoxolone methyl	The CARDINAL trial in 2017 involved 157 Alport syndrome people living with Alport syndrome. Initially promising, but a high number of serious adverse events, treatment discontinuation, and non-significant results in post-trial analysis [51]. (Clinical Trial Number NCT03019185)

Adults living with Alport syndrome should be encouraged to regularly visit https://clinicaltrials.gov**,** type 'Alport syndrome' into the search engine, and look for trials that are occurring in the UK. There is clear contact information available for each trial and people living with Alport syndrome should be encouraged to make inquiries if they think they might wish to participate or for more information.

One route to treat Alport syndrome is to combine treatments that collectively delay, or even permanently delay, the onset of kidney failure. An important new treatment with potential in Alport syndrome already in clinical use for chronic kidney disease is SGLT2 inhibitors (sodium-glucose-like 2-channel inhibitors). Dapagliflozin should be prescribed as an add-on to the highest tolerated dose of ACE inhibitor (or ARB) unless contraindicated or in place of these treatments if both are not tolerated. Recipients must currently have an estimated glomerular filtration rate (eGFR) of 25 mls/min/1.73 m^2^ to 75 mls/min/1.73 m^2^ at the start of treatment and have a urine albumin-to-creatinine ratio (uACR) of 22.6 mg/mmol or more (or type 2 diabetes). It should be noted that this is a UK guideline and is likely to differ between countries and change with time.

DAPA-CKD [52, 53] A multi-center, international, randomised, double-blind, placebo-controlled, clinical trial which examined the efficacy of a class of diabetic drug, SGLT2i, in this case, Dapagliflozin, in slowing the progression of CKD. Several trials looked at SGLT2i and diabetic kidney disease, where it has been shown to be effective. The DAPA-CKD trial included over 4000 patients with kidney disease and proteinuria, both with diabetes and kidney disease from other causes, of which Alport syndrome met the inclusion criteria. The trial results demonstrated that SGLT2i is effective at slowing the progression of kidney disease in both diabetic and non-diabetic CKD patients. It is therefore proposed that SGLT2i would be effective in Alport syndrome [54]; however, it is not known how many patients with Alport syndrome were included in the DAPA-CKD trial specifically, and no separate Alport syndrome trial has been conducted thus far. Upcoming trials using SGLT2i will hopefully answer this question. Similarly to DAPA-CKD, the EMPA-KIDNEY trial showed equally optimisitic results with Empagliflozin (an SGLT2-inhibitor) and included 6609 people with CKD of which many people living with Alport syndrome were included although Alport-specific data disappointingly remains unpublished [55]. A new study has shown that Empagliflozin reduces the toxic accumulation of lipids in podocytes and improves kidney function and albuminuria, and prolongs survival in experimental models of Alport syndrome [56].

### Support for those living with Alport syndrome

Alport syndrome is a complex condition and the isolation of living with a rare disease can be a huge challenge, especially when a person’s GP or Nephrologist may only see 1–2 cases of Alport syndrome in their career. Due to both the presence of disease modifiers (genetic, clinical, and environmental) and the X-linked nature of one form of the inherited disease, every case of Alport syndrome, even in the same family, can be very different and makes predicting the outcomes and timing difficult, worsening the feeling of isolation and uncertainty. To combat this, it is essential that doctors and those living with Alport syndrome work it out together. Doctors must refer patients and their families to the support available through their local/national organisations supporting people living with Alport syndrome, other kidney support groups, organisations, and international workshops on Alport syndrome, so they can connect with others going through similar challenges.

See Appendix Table 6 ‘Support for patients with Alport syndrome’.

## Conclusions

Alport syndrome in adults is an important cause of kidney disease, and there is often a family history of kidney disease and potentially deafness and eye problems too. Diagnosis by genetic testing determines the type of inheritance of Alport syndrome and allows early treatment of people living with Alport syndrome and identifies their relatives that may have Alport syndrome too. Treatment with the highest tolerated dose of an ACE inhibitor (or an Angiotensin Receptor Blocker (ARB)) is often effective in delaying kidney disease but does not provide a cure. Newer therapies are currently in clinical trials and it is important to keep up with the latest research on treatments.

See Appendix Table 7 (key messages regarding Alport syndrome).

See Appendix Table 8 (glossary of terms).

## Figures and Tables

**Fig. 1:**
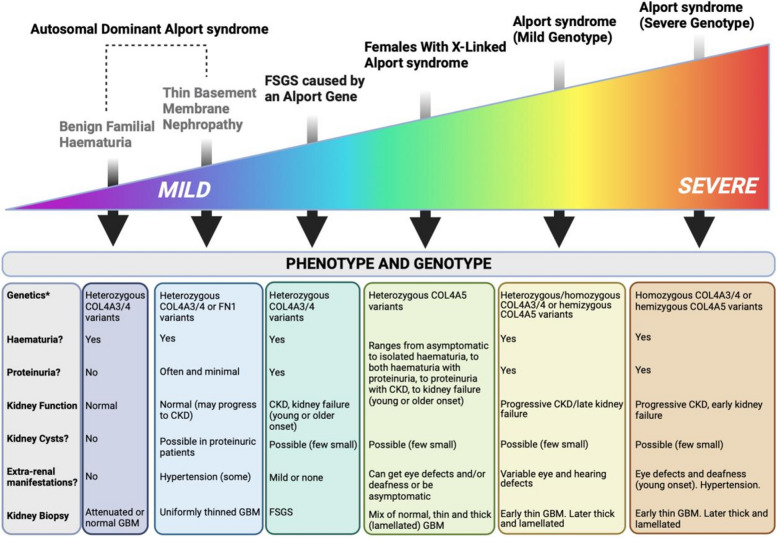
The conditions that now fall under the term ‘Alport syndrome’ and their characteristics, positioned according to their predicted clinical severity in relation to each other (please note that some individual cases may differ in their predicted severity). Focal segmental glomerulosclerosis (FSGS), glomerular basement membrane (GBM), chronic kidney disease (CKD), blood in the urine(Haematuria), protein in the urine (Proteinuria), and kidney failure (KF)

**Fig. 2:**
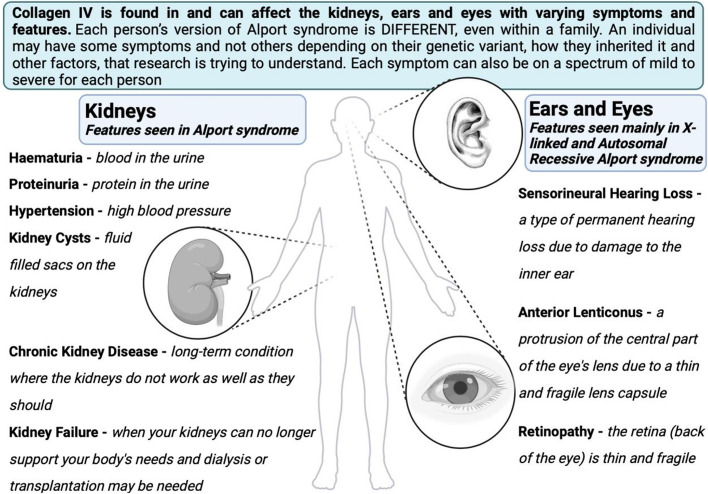
Clinical features that can be found in Alport syndrome

**Fig. 3:**
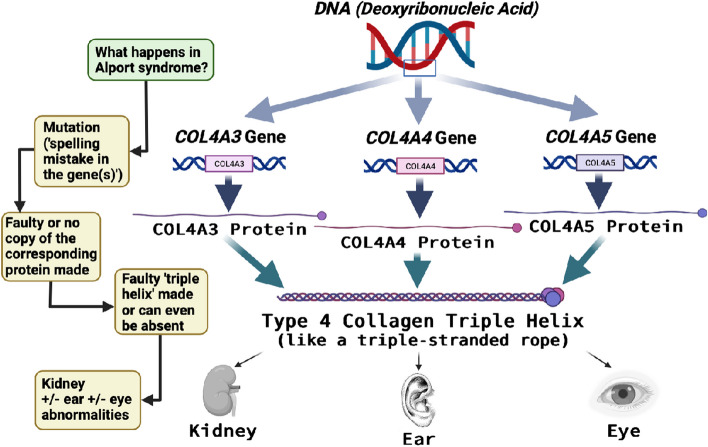
Collagen IV and how the genes instruct the making of collagen IV proteins

**Fig. 5:**
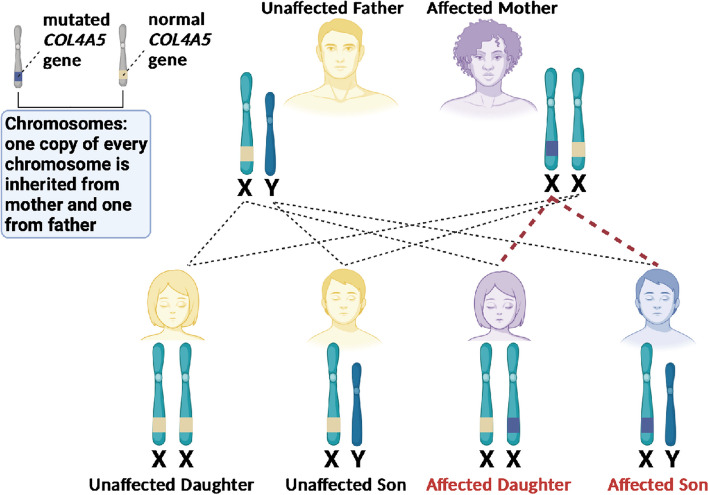
X-linked pattern of inheritance seen in Alport syndrome with COL4A5 variants

**Fig. 6:**
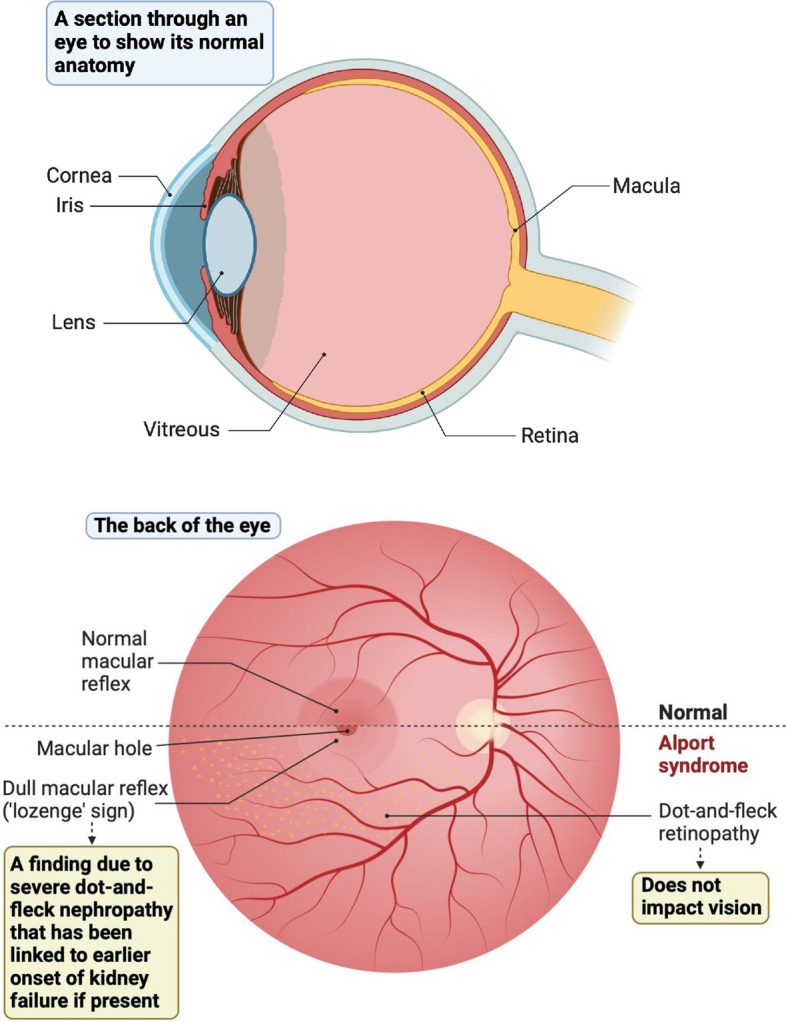
Basic anatomy of the normal eye (top image). Changes to the retina (the back of the eye) in Alport syndrome (bottom image)

**Fig. 7:**
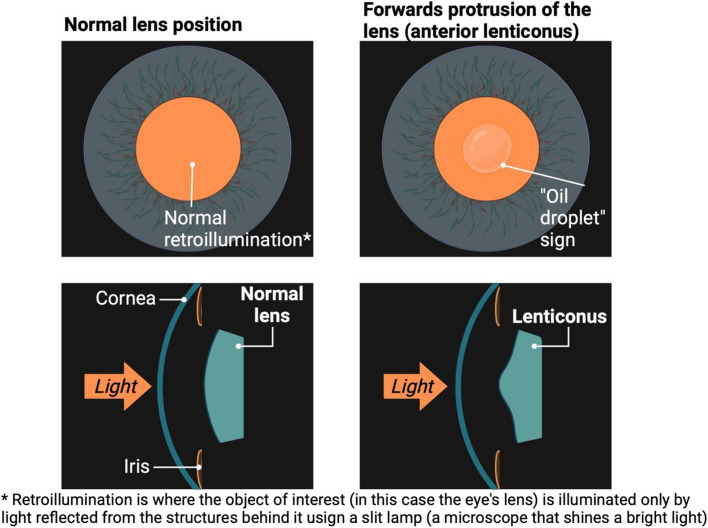
The ‘oil droplet’ sign and anterior lenticonus in Alport syndrome

**Fig. 8:**
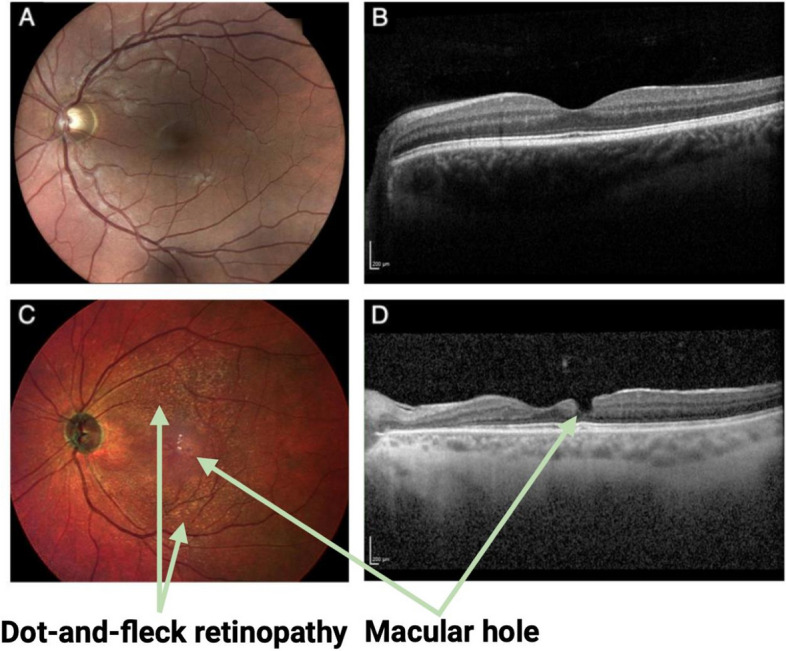
Face-on colour (left) and cross-sectional infrared (right) images of the central retinas of two male patients, both around 35 years of age, with Alport syndrome. The images show the retinal structure of a patient with a healthy retina (**A**, **B**) and another patient with dot-and-fleck retinopathy and macular hole formation (**C**, **D**)

## Data Availability

Data sharing is not applicable to this article as no datasets were generated or analysed during the current study.

## Appendix

### Getting a diagnosis of Alport syndrome as an adult

#### Patient handout

Alport syndrome is a genetic condition that can run within families. It has a large spectrum of symptoms ranging from mild to severe so it is very different from person to person even within a family. In its most severe form, Alport syndrome can cause kidney failure, deafness and eye abnormalities.

This handout is a summary of diagrams and explanations for adults living with Alport syndrome and their families to help better understand the condition. Please refer to Table 8 at the end of the handout which provides a glossary of terms and their meanings.

##### The spectrum of Alport syndrome symptoms and their severity

Below is a diagram that summarises all of the conditions that fall under the term ‘Alport syndrome’ and how severe the symptoms can be in relation to each other. Please note that some individual cases may be milder or more severe than predicted.

##### The severity of Alport syndrome is determined by the genetic variant (mutation)

Alport syndrome can be mild or severe for a number of different reasons but the type of genetic variant (gene ‘spelling mistake’) causing the condition can be a large factor. Every patient should ask for a copy of their genetic report from their doctor so that they know what type of gene variant they have that is causing their condition. Each genetic variant has a different effect on the protein it makes. In Alport syndrome, usually, one of three different genes that make collagen IV proteins in the kidney, eyes, and ears have a variant or ‘spelling mistake’.

**Table 5:** Different types of genetic variants have an effect on protein production

Variants having a milder effect	Variants having a substantial effect on the protein and associated with more severe disease
Missense or nonsense	Trucations
Splice site	Frameshifts
Small insertions and deletions	Large deletions/rearrangements
Small deletion-insertions	

##### How does Alport syndrome affect the body?

We must not ignore Leiomyomatosis which can occur although is extremely rare. This condition refers to smooth muscle tumours in various parts of the body and occurs particularly in the oesophagus (food pipe), tracheobronchial tree (large airways), and female genital tract (uterus). This is inherited in an X-linked pattern and occurs in rare cases of X-linked Alport syndrome. The exact relationship between Alport syndrome and Leiomyomatosis is not fully understood but research is looking into this. Leiomyomas can be surgically removed if causing symptoms [13].

HOW IT WORKS: What do the kidneys do?

Most people have two kidneys, which filter the blood and help get rid of waste products in the form of urine. Each kidney is made up of around 1 million units, called ‘nephrons’. Each nephron can be subdivided into two important units; a filtration unit called the ‘glomerulus’, and a fine-tuning section called the ‘tubule’. At any time, the kidneys handle approximately 20% of the blood flow within the body. This blood passes through the glomerulus and is filtered, via the glomerular basement membrane. This allows waste products and fluid to pass out into the tubule. The tubule then works to reclaim electrolytes and small molecules which the body needs, leaving behind waste products, which exit the body in the urine. A normally functioning basement membrane within the glomerulus is essential to this process. The glomerular basement membrane (GBM) is a complex structure and is a key component of the walls of the small blood vessels (capillaries) that make up the glomerulus. Collagen IV is made up of 3 ‘chains’ (A3, A4, and A5) which form a ‘triple helix’ structure like a twisted ‘rope’ made up of three different strands [57]. These chains act to strengthen and hold the glomerular basement membrane (GBM) together [58]. A normal and fully functioning basement membrane allows small waste molecules to pass out from the bloodstream into the tubule, and also prevents larger molecules such as proteins and red blood cells, from being lost from the body.

The kidneys also work to regulate fluid and acid balance in the body, control blood pressure and maintain adequate haemoglobin levels. Haemoglobin is one of the ingredients of blood which, if low, causes anaemia [59]. If kidneys do not work well, it can cause low haemaglobin and anaemia which can require treatments such as Erythropoietin injections and/or iron tablets or injections to boost haemoglobin to normal levels again.

HOW IT WORKS: The kidneys in Alport syndrome

People with Alport syndrome have spelling mistakes (sometimes referred to as variants or mutations) in the genes that code for one of three chains that make collagen IV proteins in the body (Fig. 3). These three genes are called COL4A3, COL4A4, and COL4A5. Collagen IV is a major structural component of the thin lining that supports the cells and tissues (basement membranes), specifically those of the kidneys, ears, and eyes [60].

Deoxyribonucleic acid (DNA) (our genetic code) is comprised of genes that encode proteins in the body. When there is a genetic variant (coding change or mistake) in one of our genes, it can make a faulty or dysfunctional protein. This faulty or dysfunctional protein can cause disease. In Alport syndrome, one or more of the three collagen IV genes is faulty and this causes a structural problem in the COL43/4/5 protein complex that builds the collagen IV ‘triple helix’. This triple helix is found in the kidney glomeruli (kidney filters), the ears, and the eyes, so when the collagen IV genes are faulty, problems occur in these parts of the body in Alport syndrome.

In people with Alport syndrome, the glomerular basement membrane (in the kidney) is initially thin and can develop microscopic breaks or tears in the membrane that allow blood cells to leak into the urine. Often the first sign of Alport syndrome is the presence of microscopic or ‘invisible’ blood in the urine, seen only by testing urine with a dipstick. Blood in the urine is often present in childhood [12]. When the person has an infection—a virus or bacterial infection—their kidneys ‘leak’ more blood causing the brown colour. The ‘cola-coloured’ urine is caused by ‘visible’ blood in the urine (macroscopic haematuria). In babies, this is what causes the ‘brown-looking’ nappies.

The cells within the microscopic kidney filters (glomeruli) respond to abnormal collagen IV by laying down other proteins, leading to the thickening of the glomerular basement membrane, and stopping it from being able to keep protein out of the urine. This results in proteins such as albumin also being found in the urine on dipstick testing. Over the years, this process worsens and scarring (fibrosis) occurs within the kidneys, leading to heavy protein leaks into the urine. The scarring and fibrosis get in the way of kidney function causing chronic kidney disease (CKD) and high blood pressure. High blood pressure is called hypertension [59, 60](Fig. 4).

A few people with Alport syndrome may have cysts on both kidneys; however, these are usually small and do not cause symptoms. When kidney function declines or underlying kidney disease is suspected, a kidney ultrasound scan can help to identify cysts. A kidney ultrasound helps to look at the structure of the kidneys.

##### How is Alport syndrome inherited?

Alport syndrome has different modes of inheritance depending on the specific type that is running in a family. Table 2 summarises the different types of Alport syndrome and how the condition can be passed on in a family.

**Table 6:** Modes of Inheritance in Alport syndrome

Inheritance pattern	Prevalence	Gene Affected	Number of disease-causing variants	Collagen IV α Chain Affected	Biological Sex	Who inheritance is from
Autosomal dominant Alport syndrome	1 in 100 at least	COL4A3 or COL4A4 (both on chromosome 2)	One	α3 or α4	Male and Female	Inherited from father or mother
X-linked Alport syndrome	1 in 2300 at least	COL4A5 (chromosome X)	One	α5	Male and Female	Men inherit from their mother (unless a de novo variant) Women inherit from their father or mother
Autosomal recessive Alport syndrome	Much rarer	COL4A3 or COL4A4 (both on chromosome 2)	Two in one gene only	α3 or α4	Male and Female	Inherit from both father and mother (one copy of variant from each)
Digenic	Unknown	Any two of COL4A3, COL4A4, COL4A5	Two different genes	α3, α4, α5	Male and Female	Inherited from one parent or from both

*Adapted from Judy Savige, IPNA Calgary 2022 [3, 4]

In X-linked Alport syndrome, a woman may pass the condition onto her sons or daughters and has a 50% chance of passing this onto any child regardless of sex. A man will only pass the condition to his daughters, never his sons, as he has to pass on his Y chromosome to have a son but all of his daughters will be affected. Boys inherit their X chromosome from their mother but in 15% of cases, the Alport syndrome is ‘new’ (de novo) in the family and has developed ‘spontaneously’ in a person rather than being passed down so, in this case, an affected boy’s mother should have genetic testing to see if she has the condition too.

HOW IT WORKS: How is your hearing affected in Alport syndrome?

The medical term for hearing loss in Alport syndrome is 'sensorineural deafness' and is helped by the use of hearing aids. Not everyone develops hearing loss with Alport syndrome but all people with Alport syndrome should be seen by a hearing specialist (Audiologist). Hearing loss can affect how the brain is able to function. Wearing hearing aids will help retain the brain’s ability ‘to hear’. It is very important to get hearing aids early to protect the brain’s function and your brain’s ability to work well with a hearing aid. This is especially vital for children so that their learning and development are not affected.

HOW IT WORKS: How are your eyes affected by Alport syndrome?

People with Alport syndrome do not typically have problems with their eyesight. A few people with Alport syndrome may have difficulty focussing and may need stronger prescriptions for glasses/spectacles over time. Changes happen in the eyes of people with Alport syndrome because the lining that supports cells and tissues (basement membranes) in the cornea, lens, and retina of the eye are more fragile (Fig 6). Classical eye findings in Alport syndrome can help diagnose the condition, but not having eye abnormalities does not rule out a diagnosis of Alport syndrome. Some people appear to have mild features and some appear to have more severe symptoms.

**Table 7:** Checklist of eye signs to take to your Opthalomologist or Eye Surgeon. Here is a checklist for people living with Alport syndrome to take to their Optician or Eye Surgeon, summarising the range of changes that can be seen in the eye in Alport syndrome. The symptoms of many of these conditions overlap, so if there is any concern, they should seek the opinion of an eye specialist

Diagnosis	Symptoms	Specialised tests	Treatment	Comments
Myopia/short- sightedness	Blurred vision, difficulty seeing in distance, clear when close	Refraction by Optician	Spectacles	If the prescription is ‘irregular’, get further testing by an eye surgeon you may have lenticonus
Recurrent corneal erosions	Sudden sharp pain, watering, sensitivity to bright lights, usually on walking	Slit lamp examination by an optician or ‘eye casualty’ when symptomatic using fluorescein	Lubricating eye drops if frequent, ideally a viscous ointment at night before bed	Settles within 1–2 days, if the eye is very red, must exclude infection
Posterior polymorphous dystrophy	Blurred vision	Detailed slit lamp examination, endothelial examination by eye surgeon	Nil initially, if severe may benefit from deep lamellar corneal grafting	Very rare finding
Anterior lenticonus or rarely posterior lenticonus	Classic Alport syndrome eye finding, a conical deformity of the shape of the lens surface. Blurred vision, progressive short-sightedness not correctable with spectacles	Slit lamp examination, Scheimpflug photography, pentacam imaging, wavefront analysis by eye surgeon	Lens replacement surgery	Lenticonus is a specific finding in Alport syndrome, > 90% of lenticonus seen by eye surgeons is caused by this condition
Cataract	Blurred vision, haze, and glare	Slit lamp examination by eye surgeon	Cataract extraction and intra-ocular lens implantation	This may relate to steroid use after a kidney transplant
Retinal flecks	A finding typical of Alport syndrome does not appear to have symptoms	Fundus examination by slit lamp biomicroscopy	None	White spots at the macula and the peripheral retina—not macular degeneration, also seen in carriers
Macular ‘lozenge’	No symptoms in early disease	OCT scan of the macula by an eye surgeon (ideally a retinal specialist)	None	The natural history and late findings in older people are unknown
Giant macular hole	Blind spot in central vision	OCT scan by a retinal specialist	Vireo-retinal surgery (very specialised eye surgery)	Rare

Source: Mr. Moin Mohamed and Dr Omar Mahroo, Moorfields Eye Hospital, London, UK

##### Symptoms and signs that could indicate a diagnosis of Alport syndrome.

HOW IT WORKS: Getting a diagnosis of Alport syndrome

**Table 8:** What symptoms and signs indicate a diagnosis of Alport syndrome?

Persistent blood in the urine (microscopic haematuria) especially when there is a family history of blood in the urine or kidney issues
Protein in the urine (Proteinuria) or kidney failure when there is no other obvious cause
Cases in the family of ‘IgA nephropathy’ (IgAN). IgA nephropathy may coincidentally co-exist with Alport syndrome and this can look like a family history of IgAN
Cases of focal segmental glomerulosclerosis (FSGS)—especially if this condition does not respond to steroid treatment or if there are other possible cases in the family
Hearing loss or eye features, such as fleck retinopathy
Kidney cysts should make one think of Alport syndrome, especially if PKD1/2 variants (which cause autosomal dominant polycystic kidney disease) are not found

Early diagnosis is important as most early treatments of Alport syndrome focus on reducing the pressure within the glomeruli and therefore reducing the level of protein leakage; this is thought to slow the process of kidney scarring (fibrosis), thus slowing the decline in kidney function in most patients. This is done by giving a type of blood pressure medicine called an angiotensin-converting enzyme inhibitor (ACE-inhibitor (ACE-i)) [61]. ACE-i drugs include Lisinopril, Perindopril, and Ramipril which are common tablets for high blood pressure. If there are side effects such as a persistent dry cough, an angiotensin receptor blocker (ARB) can be used instead. These families of medicines work by reducing the pressure across the glomerular basement membrane, reducing protein leak, and delaying the onset of scarring (fibrosis) in the kidney. Treatment with the highest tolerated dose of this therapy can delay the onset of kidney failure by anywhere from 3 to 40 years [62].

A specialist kidney doctor (nephrologist) will assess symptoms and investigate the possible cause(s). A diagnosis is made using clinical symptoms and signs, family history, physical assessment, blood and urine analysis, and genetic testing. Genetic testing is the international standard to diagnose Alport Syndrome [1]. Genetic testing is very useful, both to confirm the diagnosis in a person and to find out the underlying pattern of inheritance which may or may not have implications for other members of their family. Once a variant is identified, testing should be offered to other family members to find out if they inherited the same variant and require regular checkups at the clinic. In the future, certain treatments will be available for specific genetic variants. So it is vital a person fully understands the full name and their type of genetic variant. It is therefore important that those with Alport syndrome get a copy of their genetic test report. In the UK, this is requested from their doctor or they can apply to the hospital’s ‘Access to Medical Records Team’ who can provide the genetic test, providing it is in their medical records.

In addition, or as an alternative to genetic testing, sometimes a kidney biopsy may be done. A kidney biopsy is a routine test that takes a small sample of kidney tissue which is then sent to a specialist (pathologist) who looks down a microscope to analyse the tissue to help diagnose Alport syndrome.

If you have a family history of Alport syndrome, you and your relatives may be offered genetic testing. Urine dipstick screening for blood in the urine (haematuria) is not very sensitive or accurate, so genetic testing is recommended as it is much more accurate.

If you do not have a family history of Alport syndrome but have problems with chronic kidney disease (CKD), blood or protein in the urine, and high blood pressure, there are several conditions, including Alport syndrome, that could cause these symptoms and your doctor may arrange a kidney biopsy to help identify the cause, as well as request a genetic test. Genetic tests are the reliable means of testing for genetic conditions of the kidney such as Alport syndrome.

Genetic testing can take weeks to months for results to come back. This is because there are many steps to complete such as extracting and purifying the DNA, performing quality control checks, sequencing the DNA, and to analysis to look for variants. These are very complex processes.

##### What can I do to look after myself if I have Alport syndrome?

Currently, there are no curative treatments for Alport syndrome. However, the vibrant international research community is developing new treatments that will come into clinical practice. It is really important for families living with Alport syndrome and their nephrologists to review and understand the latest clinical guidelines, which are typically published after each of the international workshops on Alport syndrome [1].

One route to treat Alport syndrome is to combine treatments that collectively delay, or even permanently delay, the onset of kidney failure. The best treatment we have for this is an ACE inhibitor (or angiotensin receptor blocker (ARB)) and we can often also add in a drug called an SGLT2 inhibitor (e.g. Dapagliflozin). Some people need treatment once they are diagnosed (depending on the type of Alport syndrome) and others will be started on treatment if they develop proteinuria or high blood pressure or if their kidney function starts to decline. If kidney failure develops, a person with Alport syndrome should be eligible for a kidney transplant if there is no other contraindication and the outcome is usually very good. (Table 9)

**Table 9:** HOW IT WORKS: Optimising your physical and mental health in Alport syndrome

What can I do for my health if I have Alport syndrome?
**Lifestyle**
Eat a diet low in salt (sodium chloride) and low in ultra-processed food. The ‘DASH’ diet, a ‘plant-based’ diet, and the ‘Mediterranean’ diet all have the most evidence for health benefits in kidney conditions A normal protein intake (0.6–0.8 mg/kg/day) is recommended. Protein shakes and/or excess protein should be avoided as this can damage kidneys further
Avoid smoking and excess alcohol
Avoid taking non-steroidal anti-inflammatory drugs (NSAIDS) such as Nurofen, Ibuprofen, Naproxen, and Diclofenac as these can be harmful to your kidneys in Alport syndrome
Move and/or exercise regularly with an emphasis on cardiovascular exercise for example walking fast, cycling, running, or swimming. Please note that bodybuilding can be harmful
Having a healthy weight will make you more likely to be eligible for a kidney transplant and prevent a more rapid decline in your kidney function
Prioritise good quality sleep and limit stress in any way that works for you
Check your blood pressure regularly (you can buy a home monitor or do this via your GP practice) and send your doctor the results at each visit or if the readings become higher
Look after your mental health by seeking social connection, helping others, adopting methods of relaxation and mindfulness such as mediation/journaling and engaging in creative and fun activities
**Health care**
Attend your kidney clinic appointments to help you manage any kidney-related symptoms (and necessary diet) as kidney function progresses, cardiovascular risk and prepare you for kidney transplantation if you need it
Attend any screening appointments you might be referred for (e.g. hearing and eye screening) so you can get information, support, and treatments if needed and continue to do this on a regular basis whether you need hearing aids and spectacles/glasses or not
It is very important to wear hearing aids as soon as there is any sign of hearing loss to protect your higher neural pathways and the function of the rest of your brain. Also, try to avoid very loud noises such as through headphones/near speakers to protect your hearing from further loss
It is also very important to wear spectacles/glasses at the earliest signs of visual focus issues to protect your higher neural pathways and brain function
We recommend regularly checking the website; https://clinicaltrials.gov and typing Alport syndrome into the search engine to find out what clinical trials are or are going to be recruiting. Contact details are clearly available if you have questions or if you wish to consider taking part
**Family planning**
Both women and men should discuss family planning with your kidney doctor early so that preparations can be made to ensure a safe pregnancy for mother and baby
It is helpful to have a genetic test before a person with Alport syndrome intends to conceive. Talking with a genetic counsellor about the risks for children and all available options is recommended *including prenatal diagnosis and pre-implantation genetic testing if you might wish to consider these*
*ACE inhibitors (and potentially other drugs you might be taking) must be stopped prior to conceiving as they can cause malformations in the developing foetus*

##### What support is available to me if I or my family have Alport syndrome?

**Table 10:** Support for patients with Alport syndrome

Local renal unit	Your local renal unit can be contacted anytime. You can get information, support from staff and your questions answered that relate to your individual care. Some units have experts who can help with diet, psychological support and social services
National organisations supporting people living with Alport syndrome	These organisations vary in size and focus from country to country. They have a huge amount of resources, news, and support available for people living with Alport syndrome, carers, doctors, and researchers. They can answer your questions and give you information on Alport syndrome, local support groups, clinical trials, treatments, social events, information days, and fundraising. See their websites and social media: Australia: Alport Foundation of Australia—www.alport.org.au, contact email info@alport.org.au Belgium: AIRG Belgique—airg-belgique.org China: Chinese Alport Syndrome Parents Organisation—WeChat, contact email hello@henizaiyigi.com Germany: Alport Selbsthilfe—www.alport-selbsthilfe.de, contact email Vorstand@Alport-Selbsthilfe.de France: AIRG France—www.airg-france.fr, contact email airg.permanence@orange.fr Israel: Alport Foundation Israel—Facebook Italy: A.S.A.L., Associazione Sindrome di Alport—www.alport.it, contact email informazioni.asal@gmail.com Macedonia: contact email gordana_david@yahoo.com Spain: AIRG España—www.airg-e.org, contact email info@airg-e.org Switzerland: AIRG Suisse—www.airg-suisse.org, contact email info@airg-suisse.org UK: Alport UK—alportuk.org, contact email info@alportuk.org, follow @alportuk USA: Alport Syndrome Foundation—alportsyndrome.org, contact email info@alportsyndrome.org, follow @alportsyndromefndn The Netherlands: Nierpatiënten Vereniging Nederland (NVN)—www.nvn.nl, contact email secretariaat@nvn.nl Other countries: contact Alport Syndrome Alliance—alportsyndromealliance.org, contact email workshops@alportsyndromealliance.org
Alport Warriors	A closed Facebook group which is moderated byAlport UK—families and individuals from all over the country and internationally connect to share the key questions and issues they face every day living with Alport syndrome. There is always someone with practical tips and experience to share. See how student Joseph McLean lives with Alport syndrome: https://youtu.be/4bz5-tK6m6w
*Alport Avengers*	A group of young adults (18–35-year-olds) living with Alport syndrome who provide each other with help around the key time that Alport syndrome impacts their lives most. They help each other practically and thoughtfully through tough times and meet socially on a regular basis. Talking about their condition with their peers is sometimes the only time they feel ‘normal’
*Don’t Wait Fund*	A fund set up by patient and filmmaker Sam Clarke who cycled 4000 miles around Europe—for parents, people living with Alport syndrome, and siblings to apply for a grant to take up a new activity and/or buy a piece of equipment. https://youtu.be/7jQUSCHoQMc. Here’s how to apply: http://www.alportuk.org/support-alport/dont-wait-fund#
Alport social weekends and Alport information days	Check out the events in a specific country by going to the websites for the specific country listed above. Events are hosted to engage those who are newly diagnosed and connect them with others in similar situations and hear about the latest research. People living with Alport syndrome organise the events withthe local clinical teams and make sure all age groups get their questions answered and have fun and get to know the cities they visit. Young patients in Manchester, UK share their experiences: https://www.youtube.com/watch?v=77p7nzKz6nc
International Experts	International experts are available to explain the complexities of Alport syndrome—for both clinicians and people living with Alport syndrome
Peer Support	A chat on the phone with someone who knows what it feels like to live with Alport syndrome can be life-changing. Connect to local or national services for grants, psychosocial support, or someone nearby
International Alport syndrome workshop	Get involved in research and join an International Alport workshop. For those interested in science and research and travel, Alport UK organise a unique programme of international workshops that bring together the Alport Syndrome Alliance—a global network of inspiring people living with Alport syndrome, clinicians, and researchers developing new treatments and knowledge. As part of a wider mental health programme, we encourage young adults to travel abroad and build their confidence to ask questions about their condition and contribute to research discussions. Highlights from Siena workshop in Italy: https://youtu.be/QH8mDTmKaVU
Health Talk	This website is a hub where thousands of people have shared their experiences on film of what it is like to have a health condition in addition to explanations from experts. There are many on Alport syndrome. See website https://healthtalk.org (see Alport syndrome stories)

##### What are some key messages about Alport syndrome?

**Table 11:** Key messages regarding Alport syndrome

Alport syndrome is a genetic condition that can cause kidney failure and/or deafness and/or visual features but this is not the case for everyone with Alport syndrome as the clinical condition is extremely variable
Alport syndrome is the most common genetic kidney disease and the second most common cause of kidney failure after autosomal dominant polycystic kidney disease
Men are typically more severely affected than women in X-linked Alport syndrome but women with X-linked disease can also get early onset kidney failure so they should be closely monitored
There are other genetic subtypes of Alport syndrome and genetic testing allows precise diagnosis which allows for better family screening, a better understanding of the potential outcomes, better access to clinical trials, and is very helpful in family planning
Treatment with the highest tolerated dose of an ACE inhibitor (or angiotensin receptor blocker (ARB)) can slow the progression of kidney disease by years. Research is fast-changing due to a vibrant global research community collaborating on new treatments and it is important to understand the latest developments that might impact people living with Alport syndrome
Most people living with Alport syndrome who develop kidney failure will be eligible for a kidney transplant, which has a good chance of success

##### Glossary of terms and their meanings

**Table 12:** Glossary of terms

Abnormal gene	There are two copies of every gene, one from each of your parents, and many genes make a corresponding protein. If there is a ‘spelling mistake’ in one copy of a gene, the other one may make enough protein so that the protein is not faulty and disease does not develop. This is why we all have thousands of mistakes or ‘mutations’ in our genes and problems never occur. However, sometimes one faulty copy of a gene is enough to make either an imbalance of the corresponding protein or the protein is misfolded. Sometimes the protein is not made at all which can result in disease
Autosomal	The gene in question is located on one of the ‘numbered’ chromosomes (1–22) and not on one of the two sex chromosomes (X or Y) and not in the mitochondrial DNA either. ‘Autosomal dominant’ means that only one of your two copies of the gene has a ‘mutation’ or ‘mistake’ which usually means it has been inherited from one parent. ‘Autosomal recessive’ is when both of your copies of the gene have a ‘mutation’ usually inherited from each of your parents
Chromosome	Thread-like structures are located inside the nucleus of cells that make up your body. They are made of tightly coiled deoxyribonucleic acid (DNA)
Collagen IV	One of the 6 types of collagen protein found in the body. Made by the collagen IV genes (COL4 genes). Collagen IV makes up the structure of basement membranes (a structure on which certain cells grown) in the body
COL4A3, A4, A5	The 3 genes which make the collagen-a3, a4. and a5 proteins which wind together into a ‘triple helix’ structure to form basement membranes in the eye, ear, and kidney
Cortex	The cortex of the kidney is the tissue that surrounds the inside of the kidney organ called the ‘medulla’. The cortex is where the nephrons (blood filtering units) begin
DNA (Deoxyribonucleic acid)	DNA is the long double-stranded molecule that carries genetic information required for the development and function of that person. It is inherited from both of your biological parents
Genetic variant	A permanent change in the sequence (‘spelling’ or ‘code’) of a person’s DNA that makes up a gene
Genotype	The genetic makeup of a person
Haematuria	There is blood in your urine. There are two types of haematuria. ‘Frank’ haematuria which is when the blood is visible and ‘microscopic’ haematuria is when this cannot be seen unless using a microscope or can be tested for with a urine dipstick which is called ‘urinalysis’. Microscopic haematuria is usually described as 1 + , 2 + , 3 + , or 4 + blood on a urine dipstick depending on how much blood is present
Heterozygous	A person has inherited one ‘abnormal’ copy of a gene and one ‘normal’ copy of the same gene i.e. they have two different versions of the same gene due to a ‘spelling mistake’ in one of the copies
Homozygous	Being homozygous for a particular gene means that you have inherited two identical versions of the same gene. This usually means that you have an autosomal recessive condition as both copies of the gene have the same ‘spelling mistake’
Medulla	The inner part of the kidney. Its main job is to control the concentration of urine
Pathogenesis	The process by which disease develops
Phenotype	Symptoms and signs that result from a person’s genotype (genetic makeup). A person’s phenotype is determined by both their genetic makeup and environmental factors
Prognosis	An opinion on the likely future outcome of the condition (how it will turn out)
Proteinuria	There are high levels of protein in the urine. This can be a sign that there is a condition affecting the kidneys. The amount of protein leaking into the urine can be measured either using a urine dipstick (described as 1 + , 2 + , 3 + , or 4 + protein) or by performing urine albumin: creatinine ratio (urine ACR) or urine protein: creatinine ratio (urine PCR)
